# An increase in myocardial 18-fluorodeoxyglucose uptake is associated with left ventricular ejection fraction decline in Hodgkin lymphoma patients treated with anthracycline

**DOI:** 10.1186/s12967-018-1670-9

**Published:** 2018-10-25

**Authors:** Matteo Sarocchi, Matteo Bauckneht, Eleonora Arboscello, Selene Capitanio, Cecilia Marini, Silvia Morbelli, Maurizio Miglino, Angela Giovanna Congiu, Giorgio Ghigliotti, Manrico Balbi, Claudio Brunelli, Gianmario Sambuceti, Pietro Ameri, Paolo Spallarossa

**Affiliations:** 1 Cardiovascular Diseases Unit, IRCCS Ospedale Policlinico San Martino, IRCCS Italian Cardiovascular Network, Genova, Italy; 2 Department of Internal Medicine, University of Genova, Genoa, Italy; 3 Nuclear Medicine Unit, IRCCS Ospedale Policlinico San Martino, Genoa, Italy; 4 Department of Health Sciences, University of Genova, Genoa, Italy; 5 Emergency Medicine Unit, IRCCS Ospedale Policlinico San Martino, Genoa, Italy; 6 CNR Institute of Molecular Bioimaging and Physiology, Milan, Italy; 7 Haematology Unit, IRCCS Ospedale Policlinico San Martino, Genoa, Italy; 8 Department of Internal Medicine & Center of Excellence for Biomedical Research, University of Genova, Genoa, Italy

**Keywords:** Doxorubicin, Cardiotoxicity, ^18^FDG-PET, Left ventricular dysfunction, Heart failure

## Abstract

**Background:**

Doxorubicin (DOX)-based chemotherapy for Hodgkin lymphoma (HL) yields excellent disease-free survival, but poses a substantial risk of subsequent left ventricular (LV) dysfunction and heart failure, typically with delayed onset. At the cellular level, this cardiotoxicity includes deranged cardiac glucose metabolism.

**Methods:**

By reviewing the hospital records from January 2008 through December 2016, we selected HL patients meeting the following criteria: ≥ 18 year-old; first-line DOX-containing chemotherapy; no diabetes and apparent cardiovascular disease; 18-fluoro-deoxyglucose positron emission tomography (^18^FDG-PET) scans before treatment (PET^STAGING^), after 2 cycles (PET^INTERIM^) and at the end of treatment (PET^EOT^); at least one echocardiography ≥ 6 months after chemotherapy completion (ECHO^POST^). We then evaluated the changes in LV ^18^FDG standardized uptake values (SUV) during the course of DOX therapy, and the relationship between LV-SUV and LV ejection fraction (LVEF), as calculated from the LV diameters in the echocardiography reports with the Teicholz formula.

**Results:**

Forty-three patients (35 ± 13 year-old, 58% males) were included in the study, with 26 (60%) also having a baseline echocardiography available (ECHO^PRE^). LV-SUV gradually increased from PET^STAGING^ (log-transformed mean 0.20 ± 0.27) to PET^INTERIM^ (0.27 ± 0.35) to PET^EOT^ (0.30 ± 0.41; *P* for trend < 0.001). ECHO^POST^ was performed 22 ± 17 months after DOX chemotherapy. Mean LVEF was normal (68.8 ± 10.3%) and only three subjects (7%) faced a drop below the upper normal limit of 53%. However, when patients were categorized by median LV-SUV, LVEF at ECHO^POST^ resulted significantly lower in those with LV-SUV above than below the median value at both PET^INTERIM^ (65.5 ± 11.8% vs. 71.9 ± 7.8%, *P *= 0.04) and PET^EOT^ (65.6 ± 12.2% vs. 72.2 ± 7.0%, *P *= 0.04). This was also the case when only patients with ECHO^PRE^ and ECHO^POST^ were considered (LVEF at ECHO^POST^ 64.7 ± 8.9% vs. 73.4 ± 7.6%, *P *= 0.01 and 64.6 ± 9.3% vs. 73.5 ± 7.0%, *P *= 0.01 for those with LV-SUV above vs. below the median at PET^INTERIM^ and PET^EOT^, respectively). Furthermore, the difference between LVEF at ECHO^PRE^ and ECHO^POST^ was inversely correlated with LV-SUV at PET^EOT^ (*P* < 0.01, R^2^ = − 0.30).

**Conclusions:**

DOX-containing chemotherapy causes an increase in cardiac ^18^FDG uptake, which is associated with a decline in LVEF. Future studies are warranted to understand the molecular basis and the potential clinical implications of this observation.

## Background

Anthracyclines are the cornerstone of many life-saving treatments, and Hodgkin lymphoma (HL) is a paradigm of a curable malignancy also by virtue of anthracycline-based chemotherapy regimens, with optimal treatment providing a 10-year disease-free survival exceeding 80% [1, 2]. Unfortunately, however, anthracyclines are cardiotoxic [3].

Anthracycline cardiotoxicity may present as left ventricular (LV) dysfunction and heart failure within months or few years after exposure to high cumulative doses of drug, especially in the case of pre-existing cardiovascular disease. Nonetheless, LV dysfunction and heart failure may also develop more insidiously, several years after treatment with only moderate doses of anthracyclines [4–6]. This type of cardiotoxicity is typically observed in patients who do not have cardiovascular risk factors or disorders at the time of chemotherapy, such as first-line treated subjects with HL, for whom the incidence of anthracycline-related LV dysfunction may be as high as one in three treated patients [7].

Predicting late anthracycline cardiotoxicity is challenging. Both biomarkers and echocardiography with speckle tracking analysis or cardiac magnetic resonance imaging have been proposed to identify individuals who may require cardiac monitoring and may benefit from early cardioprotection with drugs such as beta-blockers and angiotensin converting enzyme inhibitors [8–11]. Yet, these tools are underused in clinical practice as they require additional exams for already overwhelmed patients, need expert personnel for appropriate interpretation of the results and, in some cases, are expensive.

Whole-body 18-fluoro-deoxyglucose positron emission tomography (^18^FDG-PET) is recommended for HL staging [2, 12]. While the oncologist evaluates ^18^FDG uptake in the hematopoietic system, this exam may also offer unique information concerning the effects of anthracyclines on the heart. Impaired mitochondrial oxidative metabolism with heightened glycolytic flux is a major feature of anthracycline cardiotoxicity in experimental mouse models [13, 14]; since hexokinase, the rate-limiting enzyme of glycolysis, is also responsible for phosphorylation and intracellular retention of ^18^FDG, it is expected that myocardial uptake of this tracer increases in response to anthracycline. In fact, previous studies demonstrated a change in LV ^18^FDG standardized uptake value (LV-SUV) during anthracycline exposure [15] and it has been hypothesized that ^18^FDG-PET might detect myocardial toxicity of anthracycline very early [16]. Consistent with this literature, we recently found that ^18^FDG uptake increases in mice injected with the anthracycline, doxorubicin (DOX), as well as in patients receiving anthracycline-containing chemotherapy, with a direct correlation with a composite endpoint of subsequent cardiac alterations [17]. Here, we sought to expand these findings focusing on LV ejection fraction (EF), because even minor decreases in this parameter were shown to predict clinically relevant anthracycline cardiotoxicity [18].

## Methods

### Patient selection

This retrospective study included patients affected by HL and referred to the IRCCS San Martino Policlinic Hospital, Genova, Italy, between January 2008 and December 2016. According to the research mission of the hospital and as per approval by the local Institutional Review Board, all subjects signed an informed consent allowing the utilization of their anonymized clinical data for scientific purposes.

**Fig. 1:**
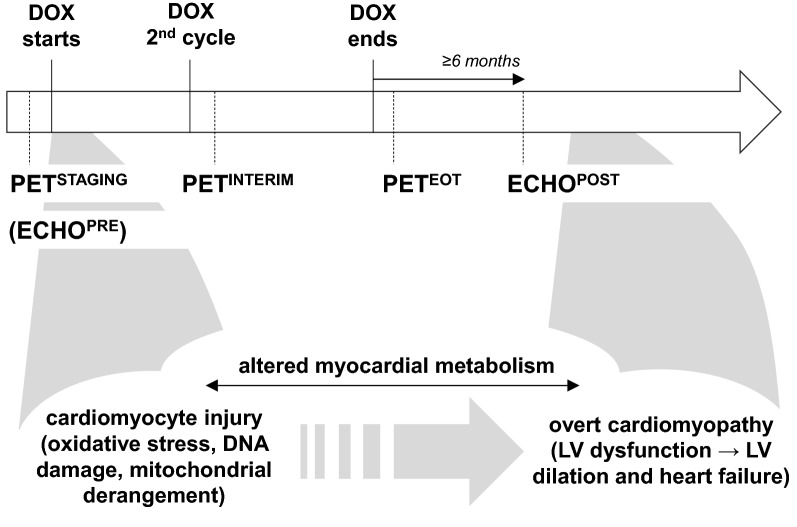
Study design and stages of anthracycline cardiotoxicity. A schematic of the timing of the ^18^FDG positron emission tomography (^18^FDG-PET) scans and echocardiograms taken into analysis is depicted in the upper panel, while the corresponding stages of anthracycline-related cardiotoxicity are presented in the lower part. DOX: doxorubicin-containing chemotherapy; ECHO^PRE^: echocardiography at baseline (available only in a subgroup of patients); ECHO^POST^: echocardiography after completion of DOX chemotherapy; PET^STAGING^: ^18^FDG-PET before treatment; PET^INTERIM^: ^18^FDG-PET after 2 cycles of doxorubicin; PET^EOT^: ^18^FDG-PET at the end of treatment; LV: left ventricular

By reviewing the medical records, eligible patients were identified based on the following criteria (Fig. 1): age ≥ 18 years; HL receiving first-line chemotherapy with DOX as part of the ABVD or BEACOPP protocols; at least three ^18^FDG-PET scans available, i.e. before treatment (PET^STAGING^), after 2 cycles (PET^INTERIM^) and at the end of treatment (PET^EOT^); no diabetes; no more than two major cardiovascular risk factors, no history of cardiac disease and normal ECG at baseline; and transthoracic echocardiography performed ≥ 6 months after DOX exposure (ECHO^POST^). Thus, by study design all subjects underwent an echocardiogram 6 or more months after chemotherapy completion. Some patients, however, received more than one post-treatment echocardiogram: in this case, the most recent one was considered (i.e. closest to the time when the retrospective analysis was carried out). When available, data of a baseline echocardiographic evaluation (ECHO^PRE^) were also taken into account.

### Nuclear imaging

^18^FDG-PET/CT scans were performed using a 16-slice Biograph 16 PET/CT hybrid system (Siemens Medical Solutions). In accordance to standard clinical practice, each patient received an intravenous bolus of 4.8–5.2 MBq/kg ^18^FDG. PET/CT acquisition started after 60–75 min, during which subjects were recommended to drink water in order to increase the urinary clearance of the unbound ^18^FDG fraction. The body was scanned from vertex to mid-thigh in arms-up position. The emission scan lasted 120 s per bed position. PET raw data were reconstructed using ordered-subset expectation maximization (OSEM, 3 iterations; 16 subsets), and attenuation was corrected using the raw CT data. 16-detector-row helical CT was performed with non-diagnostic current and voltage settings (120 kV; 80 mA), a gantry rotation speed of 0.5 s, and a table speed of 24 mm per gantry rotation. The entire CT dataset was merged with the three-dimensional PET images using an integrated software interface (Syngo; Siemens Medical Solutions).

Volumes of interest were manually drawn on the metabolically active LV myocardium and on a 2 cm-thick section of longissimus thoracis muscle at the level of 12th vertebral body. When the LV was not clearly identifiable on PET images due to the low myocardial ^18^FDG uptake, hybrid PET/CT images were used to select the volume of interest. Next, the mean SUV within the LV and skeletal muscle (SM) volumes of interest were measured (LV-SUV and SM-SUV, respectively) and normalized for the circulating ^18^FDG concentration, as estimated by the mean SUV value in a volume of interest drawn at the level of the inferior vena cava, in order to correct for the noise signal of the blood pool. The ratio between LV-SUV and SM-SUV was then calculated. These analyses were carried out by two nuclear medicine specialists with experience in ^18^FDG-PET and cardiac imaging, who were blinded to other data.

### Echocardiography

All patients underwent standard transthoracic echocardiography. LVEF was measured by means of the Teicholz formula by two cardiologists, different from those who selected the study cases and blinded to clinical and nuclear medicine data. Although a LVEF value was written in the echocardiography reports, this parameter was recalculated in order to have standardized and thereby comparable numbers.

### Statistical analysis

Statistical analysis was performed using the statistical package of R-software (ver. 3.4). Data are given as number (percentage of total), mean ± standard deviation or median (interquartile range). LV-SUV and SM-SUV values were normalized by logarithmical transformation. Comparisons were drawn by unpaired or paired Student’s t-test or repeated measures ANOVA, as appropriate, while the relationship between LVEF changes and LV-SUV was assessed by Pearson’s correlation test. The association between LVEF changes and LV-SUV was also examined in a linear regression model with age and DOX dose as covariates, since they can affect LV function. A *P* value < 0.05 was considered significant.

## Results

Sixty-five patients were eligible during the study period, but 22 did not have ECHO^POST^, leaving a sample of 43 subjects. For 26 of them (60%), ECHO^PRE^ was available.

The baseline characteristics of the 43 patients are summarized in Table 1. Mean age was 35 ± 13 years, male gender was slightly predominant and the prevalence of cardiovascular risk factors was low. Consistent with the fact that diabetes was a cause of exclusion from the study, mean fasting plasma glucose was normal at baseline (82 ± 9 mg/dL), and remained normal at PET^INTERIM^ (83 ± 10 mg/dL) and PET^EOT^ (86 ± 24 mg/dL). LVEF in the subgroup with ECHO^PRE^ was always within the normal range.

**Table 1:** Baseline characteristics of the patients included in the study

Male	25 (58%)
Age (years)	35 ± 13
Age > 65 years	0
Hypertension	0
Smoke	18 (42%)
Dyslipidemia	4 (9%)
Family history of heart disease	8 (19%)
Chronic kidney disease	0
Mediastinal RT (non-cardiac field)	6 (14%)
Doxorubicin dose (mg/m^2^)	251 ± 57
ECHO^PRE^	26 (60%)
LVEDD (mm)	47.2 ± 5.2
LVESD (mm)	28.2 ± 3.9
LVEF (%)	70.3 ± 7.1

RT: radiotherapy; ECHO^PRE^: baseline echocardiography; LVEDD: left ventricular end-diastolic diameter; LVESD: left ventricular end-systolic diameter; LVEF: left ventricular ejection fraction

**Fig. 2:**
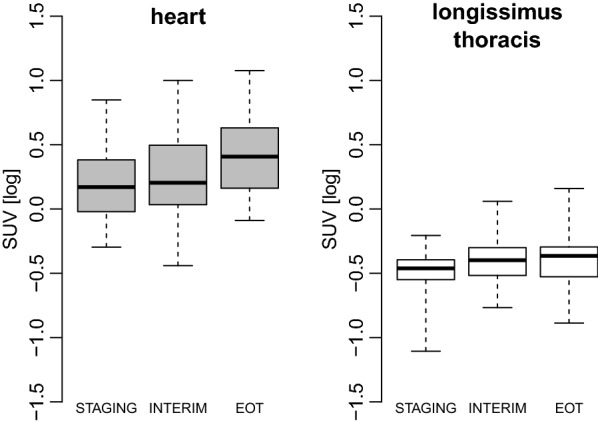
^18^FDG uptake during doxorubicin treatment in the myocardium and in the longissimus thoracis muscle. Boxes are median and interquartile ranges of left ventricular and skeletal muscle ^18^FDG standardized uptake values (SUV) at the indicated ^18^FDG positron emission tomography (^18^FDG-PET) scans. Vertical bars indicate the highest and lowest SUV at each time point. Repeated measures ANOVA *P* for trend = 0.0007 and 0.02 for SUV in the heart and longissimus thoracis, respectively. STAGING: ^18^FDG-PET before treatment; INTERIM: ^18^FDG-PET after 2 cycles of doxorubicin; EOT: ^18^FDG-PET at the end of treatment

A significant progressive increase in LV-SUV was observed over DOX treatment (Fig. 2), and this trend was confirmed in the subgroup of patients with ECHO^PRE^ (data not shown). SM-SUV also increased during chemotherapy (Fig. 2), thus no significant change occurring in the ratio between LV-SUV and SM-SUV.

ECHO^POST^ was performed 22 ± 17 (range 6–76) months after DOX chemotherapy. LVEF dropped below the upper normal limit of 53% in 3 (7%) patients, who remained asymptomatic, and mean LVEF in ECHO^POST^ was normal (68.8 ± 10.3%), as was LV end-diastolic diameter (LVEDD) (47.4 ± 5.1 mm). In the subgroup with both ECHO^PRE^ and ECHO^POST^, mean LVEF and LVEDD minimally and non-significantly changed from 69.0 ± 9.3 to 70.3 ± 7.1% (*P* = 0.43) and from 47.2 ± 5.2 to 46.9 ± 4.9 mm (*P* = 0.60), respectively.

**Fig. 3:**
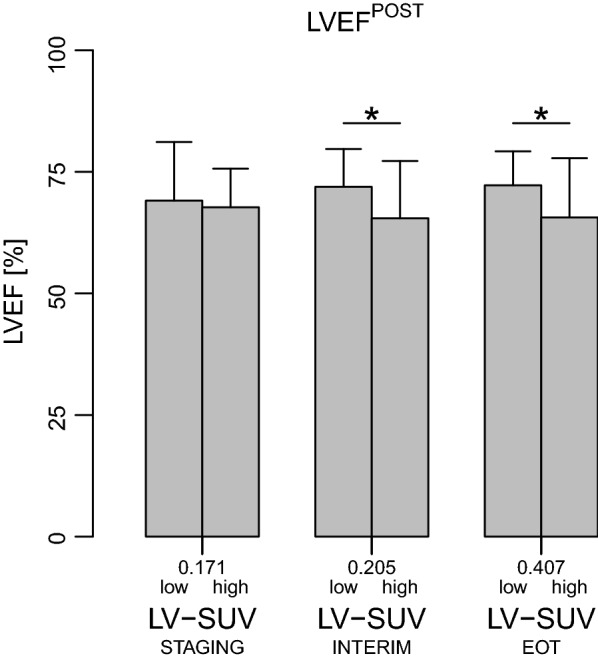
Left ventricular ejection fraction according to categories of ^18^FDG uptake in patients with echocardiography at follow-up. Left ventricular ejection fraction (LVEF), as assessed by echocardiography performed after completion of anthracycline chemotherapy, in patients with myocardial ^18^FDG standardized uptake values (LV-SUV) below or above the median value (low and high, respectively) measured at each ^18^FDG positron emission tomography (^18^FDG-PET) scan. For each PET time point, LVEF was compared between patients with LV-SUV below vs. above the median value by unpaired t-test. * indicates *P* < 0.05. LV-SUV^STAGING^: LV-SUV at the ^18^FDG-PET scan before treatment; LV-SUV^INTERIM^: LV-SUV at the ^18^FDG-PET performed after 2 cycles of doxorubicin; LV-SUV^EOT^: LV-SUV at the ^18^FDG-PET performed at the end of chemotherapy

**Fig. 4:**
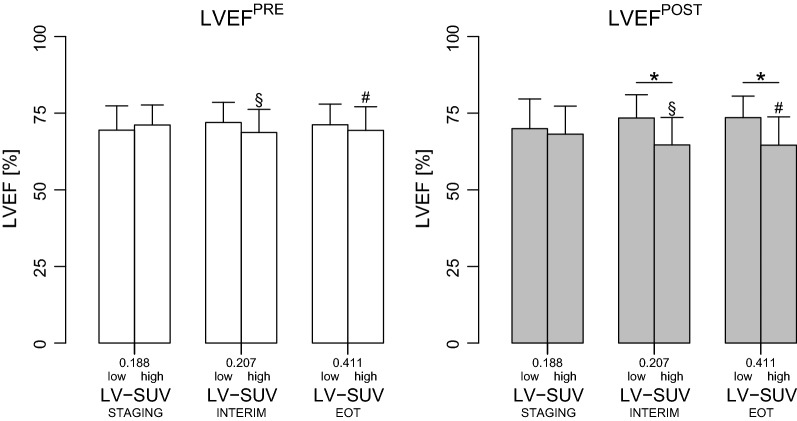
Left ventricular ejection fraction according to categories of ^18^FDG uptake in patients with echocardiography at both baseline and follow-up. Left ventricular ejection fraction (LVEF) at the baseline and follow-up echocardiography (LVEF^PRE^ and LVEF^POST^, respectively) in patients with myocardial ^18^FDG standardized uptake values (LV-SUV) below or above the median value (low and high, respectively) at each ^18^FDG positron emission tomography (^18^FDG-PET) scan. LVEF was compared between patients with LV-SUV below vs. above the median value at each PET time point by unpaired t-test and * is *P* < 0.05. LVEF^PRE^ and LVEF^POST^ for each subgroup of patients with LV-SUV below or above the median value (e.g. LVEF^PRE^ and LVEF^POST^ in subjects with high LV-SUV at EOT) were compared by paired t-test; § and # indicate *P* < 0.05 and < 0.01, respectively. LV-SUV^STAGING^: LV-SUV at the ^18^FDG-PET scan before treatment; LV-SUV^INTERIM^: LV-SUV at the ^18^FDG-PET performed after 2 cycles of doxorubicin; LV-SUV^EOT^: LV-SUV at the ^18^FDG-PET performed at the end of chemotherapy

**Fig. 5:**
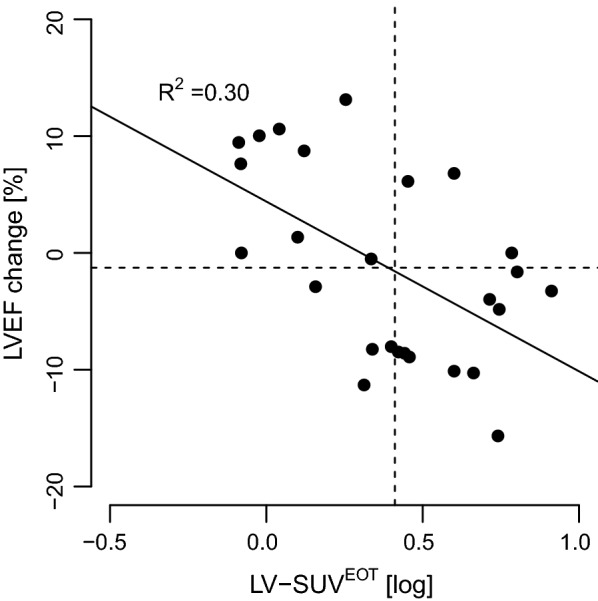
Relationship between left ventricular ejection fraction change and ^18^FDG uptake in the subsets of patients with baseline and follow-up echocardiography data available. LVEF: left ventricular ejection fraction; LV-SUV^EOT^: LV ^18^FDG standardized uptake value at positron emission tomography performed at the end of anthracycline chemotherapy

However, when patients were categorized in high and low LV-SUV by LV-SUV median value, LVEF was significantly lower in those with high vs. low LV-SUV values at both PET^INTERIM^ and PET^EOT^ (Fig. 3). This was also the case when only subjects with ECHO^PRE^ and ECHO^POST^ were considered (Fig. 4). Interestingly, LVEF at ECHO^PRE^ was similar among LV-SUV categories at any PET scan; consequently, LVEF as measured at ECHO^POST^ was significantly lower than LVEF at ECHO^PRE^ in patients who had a high LV-SUV at either PET^INTERIM^ or PET^EOT^ (Fig. 4). In addition, the difference between LVEF at ECHO^PRE^ and ECHO^POST^ was inversely correlated with LV-SUV^EOT^, the higher being LV-SUV^EOT^ the wider the decrease in LVEF (Fig. 5). The relation between LV-SUV^EOT^ and the magnitude of LVEF change remained significant after adjusting for age and dose of DOX (univariate analysis: β − 14.5, SE 4.6, *P* = 0.004; after accounting for age and DOX dose: β − 13.7, SE 4.9, *P* = 0.01).

By contrast, LVEDD was not significantly different among the high and low LV-SUV categories at any PET time point (Table 2).

**Table 2:** Left ventricular end-diastolic diameter across categories of ^18^FDG uptake at each ^18^FDG-PET scan

	LV-SUV	LVEDD (mm)	*P*
PET^STAGING^	Low	47.3 ± 5.2	0.81
	High	47.7 ± 5.2	
PET^INTERIM^	Low	46.1 ± 4..9	0.11
	High	48.7 ± 5.2	
PET^EOT^	Low	47.7 ± 4.8	0.90
	High	47.7 ± 5.1	

Left ventricular end-diastolic diameter (LVEDD), as assessed by echocardiography performed after completion of anthracycline chemotherapy, in patients with myocardial ^18^FDG standardized uptake values (LV-SUV) below or above the median value (low and high, respectively) measured at each ^18^FDG positron emission tomography (^18^FDG-PET) scan

For each PET time point, LVEDD was compared between patients with LV-SUV below vs. above the median value by unpaired t-test

LV-SUV^STAGING^: LV-SUV at the ^18^FDG-PET scan before treatment; LV-SUV^INTERIM^: LV-SUV at the ^18^FDG-PET performed after 2 cycles of doxorubicin; LV-SUV^EOT^: LV-SUV at the ^18^FDG-PET performed at the end of chemotherapy

## Discussion

The present study shows that DOX-containing chemotherapy causes an increase in ^18^FDG uptake by the normal heart, as defined by clinical assessment, which is associated with a significant decline in LVEF several months to years after treatment completion. These results substantiate an emerging literature putting attention on the effects of anthracyclines on myocardial retention of ^18^FDG-PET [15–17, 19], and raise research and practical prospects.

First, our and other authors’ work prompts the question of which mechanism underlies the higher accumulation of ^18^FDG in the myocardium exposed to DOX as compared with control conditions. Finding the answer would imply gaining insights into the pathogenesis of DOX cardiotoxicity, which is complex and still to be fully resolved [20], and possibly laying the foundations for novel strategies to avoid or mitigate cardiac injury.

DOX and other anthracyclines acutely and invariably elicit a triad of alterations in cardiomyocytes—oxidative stress, DNA damage due to topoisomerase II poisoning and mitochondrial derangement [21]. Intriguingly, however, development of gross cardiac abnormalities is delayed, in both the animal model and, to the largest extent, patients [22, 23]. Therefore, anthracycline cardiotoxicity may be schematically divided in immediate molecular and cellular events, which occur right after drug administration, and whole-organ clinically relevant perturbations, which most often appear after a temporal gap that may last months or even years (Fig. 1). The reason for this two-stage course remains elusive, and several, likely not mutually exclusive factors, such as senescence [24] or stalled autophagy [14], have been suggested.

Abnormal energy metabolism with depressed mitochondrial oxidation and enhanced lactate-producing glycolysis characterizes both the early and the late phases of anthracycline cardiotoxicity [13, 14, 23] (Fig. 1). Within this metabolic reorganization, hexokinase displays increased activity [13, 14, 25, 26]. Since this enzyme is also thought to be responsible for the phosphorylation of ^18^FDG leading to intracellular tracer retention [27], it is conceivable that the augmented myocardial uptake of ^18^FDG following DOX chemotherapy is the consequence of heightened hexokinase activity. If so, this and other recent studies [16, 17, 19, 28] may be viewed as indirect confirmation that anthracycline-initiated impairment of energy metabolism occurs in the human heart as it does in experimental models.

There may be other explanations for cardiac ^18^FDG accumulation upon exposure to DOX. Intracellular mediators other than hexokinase may mediate at least part of ^18^FDG uptake in cardiomyocytes, as it has recently been proposed for cancer cells [29]. Furthermore, the possible contribution of an increase in the expression of transmembrane glucose (and ^18^FDG) transporters [30], as well as of non-cardiomyocytes, must be taken into consideration.

Regardless of how the heart becomes more avid of ^18^FDG after anthracycline chemotherapy, this phenomenon might be exploited for cardiotoxicity surveillance.

Nowadays, LVEF monitoring by repeated echocardiography is the standard of care to detect anthracycline cardiotoxicity, but it captures only the late steps of anthracycline-elicited cardiomyopathy, when global LV systolic dysfunction develops. The ideal marker should allow the recognition of anthracycline damage much earlier, when cardiac homeostasis is profoundly disrupted but the whole heart appears normal. Both troponin, a biomarker of cardiomyocyte injury [31], and echocardiography with LV global longitudinal strain analysis, which identifies an impairment in myocardial deformation that occurs when LVEF is still preserved, have been proposed for the scope [8, 9, 11]. Nevertheless, implementation of these screening approaches in clinical practice is difficult, one main reason being that they represent further procedures added to the already high number of diagnostic tests the oncological patient undergoes, with not negligible personal and health care costs.

By contrast, ^18^FDG-PET offers the unique advantage that it is routinely performed for HL [1, 2, 32] and does not require additional radiation or exam prolongation. In our cohort, higher myocardial ^18^FDG uptake was associated with a small but significant drop of LVEF values to the very low part of the range of normality, suggesting that systematic analysis of radiotracer retention by the heart during ^18^FDG-PET done for oncological purposes may be another way to detect anthracycline cardiotoxicity. Additional work is needed to confirm this opportunistic use of ^18^FDG-PET and whether cardiac ^18^FDG accumulation may anticipate LVEF decline, since we did not evaluate echocardiograms performed at the same time of PET scans. Similarly, it has to be investigated whether and how ^18^FDG-PET can be integrated with other imaging techniques and/or circulating biomarkers.

Only a few of our patients had LVEF below the upper normal limit and there were no cases of heart failure at follow-up. Nonetheless, the study population was made of young individuals with low cardiovascular risk, for whom minor decreases in LVEF are not expected and must be deemed biologically relevant, even though clinically silent. This is even more so considering that such patients are generally believed not to be prone to cardiovascular events and therefore are not monitored in this regard. Moreover, it was shown that subjects treated with anthracyclines who initially face a small reduction in LVEF are at greatest risk of developing heart failure over time [18].

There are some limitations of this work to be acknowledged. Because of the retrospective design, LVEF was calculated from reported LV diameters. However, in the presence of normal LV size and regional kinesis like for the cases we reviewed, the Teicholz formula is reliable to estimate LVEF [7]. Second, the ^18^FDG-PET scans we analyzed were obtained without any dietary preparation of the patients, as it is routinely done in oncological practice. Even though we corrected for SM activity, it cannot be excluded that at least part of the variations in myocardial tracer uptake were due to different dietary regimens followed by the studied subjects. Prospective investigations are needed to overcome this shortcoming, by including a high-fat low-carbohydrate diet or pharmacological preparation to minimize diet-dependent LV-SUV variability. Finally, the method used to evaluate myocardial metabolism in non-dedicated ^18^FDG-PET is not yet standardized and the LV-SUV thresholds according to which patients were categorized were arbitrary. Future studies should define the optimal technical aspects and reduce inter-reader and inter-scanner variability [33].

## Conclusions

We propose ^18^FDG-PET as a tool to better characterize and monitor anthracycline toxicity on the human heart, with potential clinical implications. Further research is warranted to support this claim and expand the evidence from low-risk subjects like the ones we studied to a population that ultimately develop heart failure. From the basic science standpoint, our results reiterate the emphasis on the metabolic aspects of anthracycline cardiotoxicity.

## Abbreviations

HL: Hodgkin lymphoma
LV: left ventricle
^18^FDG: ^18^F-fluoro-deoxyglucose
PET: positron emission tomography
LV-SUV: left ventricular standardized uptake value
DOX: doxorubicin
PET^STAGING^: PET before treatment
PET^INTERIM^: PET after 2 cycles
PET^EOT^: PET at the end of treatment
ECHO^PRE^: baseline echocardiography
ECHO^POST^: follow-up echocardiography
LVEF: ejection fraction
LVEDD: LV end-diastolic diameter
CT: computed tomography
SM: skeletal muscle
